# Effect of Emotional Valence on Emotion Recognition in Adolescents with Autism Spectrum Disorder

**DOI:** 10.1007/s10803-022-05831-5

**Published:** 2023-01-13

**Authors:** Sarah J. Palmer, Adrian Fanucci-Kiss, Ella Kipervassar, Isha Jalnapurkar, Steven M. Hodge, Jean A. Frazier, David Cochran

**Affiliations:** 1 Department of Psychiatry, UMASS Chan Medical School, 55 N Lake Ave, Worcester, MA United States; 2 UMASS Chan Medical School, 55 N Lake Ave, Worcester, MA United States; 3 Department of Radiology, Mount Auburn Hospital, 777 Concord Ave, Cambridge, MA United States; 4 Eunice Kennedy Shriver Center, UMass Chan Medical School, 55 N Lake Ave Worcester, Worcester, MA United States

**Keywords:** Autism Spectrum Disorder, Theory of Mind, Reading the Mind in the Eyes, Emotional Valence

## Abstract

This study investigated how emotional valence of a perceived emotional state impacted performance on the Reading the Mind in the Eyes task (RMET) in adolescents with autism spectrum disorder (ASD) and typically developing (TD) controls. Valence of items on the RMET, Adult (RMET-A) and Child (RMET-C) versions, was first classified in a survey of 113 medical students. Adolescents with ASD (N = 33) and TD adolescents (N = 30) were administered both RMET versions. Individuals with ASD made more errors than TD controls on positive and negative, but not neutral, valence items. The difference in performance was accentuated on the RMET-A compared to the RMET-C. Both emotional valence and complexity of language contribute to RMET performance in individuals with ASD.

Autism spectrum disorder (ASD) is a developmental disorder characterized by social communication deficits and repetitive, restricted behaviors and interests (American Psychiatric Association, 2013). Research consistently demonstrates a link between ASD and difficulty with “Theory of Mind” (ToM) tasks (Harms et al., 2010; Sucksmith et al., 2013; Wallace et al., 2011). ToM is a cognitive skill that allows an individual to attribute mental states to themselves or to others (Premack & Woodruff, 1978). This skill is essential for social functioning, especially in terms of understanding and predicting another’s behavior (Baron-Cohen et al., 2001). Emotional valence refers to the direction of behavioral activation associated with emotion, either toward (pleasant emotion) or away from (unpleasant emotion) a stimulus (Lane et al., 1999) and has been hypothesized to impact the ability of all individuals to perform ToM tasks. This study seeks to further investigate the effect of emotional valence on the ability of adolescents with ASD and neurocognitively typically developing controls (TD) to perform ToM tasks.

Story-telling paradigms to assess emotion processing in ToM tasks have revealed trends of atypical emotional language use in the story narratives, emotional descriptions, and conversations among individuals with ASD (Lartseva et al., 2015). Story-based paradigms have also been used to investigate how emotional valence affects ToM. Teh and colleages (2018) asked children with ASD and TD children to narratively describe scenes in a series of pictures, and they found that negative valence increases emotional language production. This finding is consistently demonstrated in the literature which shows that negative emotional stimuli tend to activate or capture more attentional resources compared to positive emotional stimuli (Balconi et al., 2012; Charles et al., 2003; Johansson et al., 2004; Öhman et al., 2001; Rumpfa et al., 2012). Narrative production, however, is a complex task that depends on many independent factors, including language, pragmatics, memory, planning and organizational skills (Diehl et al., 2006; Teh et al., 2018). This multifactorial process introduces potential confounding due to known narrative-discourse skill deficits seen in many children with ASD such as reduced syntactic complexity, reduced pragmatic abilities and/or limited cognitive skills (Hill, 2004; Teh et al., 2018). By contrast, single-picture emotion identification tasks may avoid such potential confounds and are therefore beneficial in elucidating the effects of emotional valence on ToM performance in individuals with ASD (Teh et al., 2018).

The Reading the Mind in the Eyes Test is an internationally recognized single-picture stimuli method of assessing ToM (Baron-Cohen et al., 2001; Jankowiak-Siuda et al., 2016; Kotrla Topić & Perković Kovačević, 2019; Miguel et al., 2017; Morandotti et al., 2018). As the original version of the RMET was being revised, a child version (RMET-C) was developed to parallel the 36-item revised adult version (RMET-A). The RMET-C uses a simpler vocabulary in order to assess ToM among children and contains 28 test images, 25 of which were carried over from the revised adult version (Baron-Cohen et al., 2001).

Compared to TD children, children with ASD have consistently demonstrated difficulties with the RMET, identifying fewer mental states correctly (Baribeau et al., 2015; Demurie et al., 2011; Kaland et al., 2008). On the other hand, there has been conflicting evidence on the impact of emotional valence (i.e., positive valence such as “happy” or negative valence such as “sad”) on task performance in ASD. Several studies using facial emotion recognition have found subjects with ASD to have greater difficulty identifying emotions of negative valence, such as fear (Howard et al., 2000; Pelphrey et al., 2002), sadness (Wallace et al., 2011), and anger (Howard et al., 2000; Pelphrey et al., 2002; Wallace et al., 2011; Ashwin et al., 2006). These studies utilized various ToM tasks including the Benton Test of Facial Recognition, visual scanpath data and emotion recognition (Pelphrey et al., 2002), and the Emotional MultiMorph Task with Ekman and Friesen’s Picture of Facial Affect Series (Howard et al., 2000; Wallace et al., 2011). On the other hand, Baribeau and colleagues (2015) found that individuals with ASD only performed worse than TD children on the RMET-C on items identified as having positive valence emotions, and there was no difference in accuracy on neutral or negative valence items. We sought to further investigate the use of the RMET to evaluate the effect of emotional valence on emotion identification in ASD.

Numerous RMET studies (Baribeau et al., 2015; Fertuck et al., 2009; Koizumi & Takagishi, 2014; Scott et al., 2011) have utilized an emotional valence classification developed by Harkness et al. (2005) in which faces from the RMET-A were classified as positive (e.g. kind), neutral (e.g. relaxed), and negative (e.g. hate) valences using a 7-point scale (1 = very negative, 4 = neutral, and 7 = very positive) (Harkness et al., 2005). Stimuli with mean ratings significantly below neutral were classified as negative, and stimuli with mean ratings significantly above neutral were classified as positive. Stimuli that did not significantly differ from neutral were classified as neutral. However, these valences were determined by raters who were shown each face together with its correct label, and the stimulus valence classifications were determined using a small sample size (N = 12), potentially leading to type II classification error of positive or negative valence items as “neutral”. Compared to evaluating the valence of the eye region stimulus alone without the label, the presence of the term describing the “correct” mental state could have biased the valence assignments. Social interactions require accurately assessing mental states from facial expressions, posture, prosody, and other qualitative features without explicit verbal clues to the emotions people may be experiencing.

Consequently, determining the emotional valence of each face used in the RMET independently from any labels allows for a more refined analysis of RMET responses. In a 2011 study examining RMET-A performance in a nonclinical sample of young adults with borderline personality disorder traits, Scott and colleagues classified RMET-A images independently of labels, otherwise following the stimulus classification procedure employed by Harkness and colleagues (2005). However, a relatively small sample of 40 undergraduate students was used to rate the valence of items.

Valence classifications also remain to be established for RMET-C stimuli, as three test images are unique from those used in the RMET-A. Koizumi & Takagishi (2014) describe valences for the 28 RMET-C test images citing classifications by Fertuck and colleagues (2009) (Fertuck et al., 2009). However, it is unclear how these RMET-C classifications were made because Fertuck and colleagues (2009) used the classifications done by Harkness and colleagues (2005) which pertains only to the 36-item RMET-A. In addition, the classifications by Koizumi and Takagishi did not match those reported by Harkness and colleagues (see Appendix 1). Finally, Baribeau and colleagues (2015) derived their RMET-C valence classifications from Koizumi & Takagishi (2014) and two differing RMET-A classifications. Therefore, to our knowledge ours is the *first* study to classify RMET-C stimuli by valence using raters.

Additionally, it remains to be determined which RMET version is most appropriate for evaluating ToM in the adolescent age group, as studies thus far have not compared performance between these two versions in typically developing adolescents or adolescents with ASD. Given the importance of this stage of development for complex social emotion processing, it is critical to understand how the complexity of emotion processing affects the accuracy of emotion identification in ASD (Garcia & Scherf, 2015). In a study using the RMET-C, typically developing adolescents (age 14–16) performed less accurately than both children age 10–12 and adults (Gunther Moor et al., 2012). In a different study using the RMET-A, typically developing adolescents (age 12–14) identified expressions less accurately than adults despite controlling for verbal and working memory abilities (Vetter et al., 2013). We included both versions of the RMET in our study, as it has not been determined which version is most appropriate in for adolescents.

This study has four objectives: (1) classify each face stimulus from the RMET-A and RMET-C as an emotional valence (on a discrete scale from most negative, e.g. hate, to neutral, e.g. relaxed, to most positive, e.g. elated); (2) examine whether, compared to TD adolescents, adolescents with ASD demonstrate impaired ToM on the RMET-A and -C; (3) determine if there is a relationship between the emotional valence of RMET facial stimuli and error rate in individuals with ASD or TD; and (4) compare the responses of adolescents with ASD to TD adolescents on the two versions of the RMET to determine if the complexity of language used for label choices impacts performance.

## Methods

### Study 1. Identifying the Emotional Valence of RMET Photographs

The 37 facial image stimuli from the RMET-A and the 29 facial image stimuli from the RMET-C were classified by valence from very negative (-3 on Likert scale) to very positive (+ 3). These images include a “practice” stimulus that is the same image for both tests that is not included in the scoring algorithm. The emotional valence of each facial image stimulus used in the RMET-A and RMET-C was measured independently of the target labels in a survey of adults.

### Participants

Medical students at the UMass Chan Medical School received an e-mail with a link to an anonymous, voluntary survey. Each of the 113 participants provided written informed consent before completing the survey.

### Materials

The survey consisted of the 40 distinct eye region photographs used in the RMET-A and RMET-C. Participants were asked to rate the valence of the eye regions on a 7-point Likert scale from − 3 (very negative, e.g. hostile) to + 3 (very positive, e.g. friendly). The order of presentation was consistent with the standard order in the originally published RMET-A followed by the 3 images unique to the RMET-C (items 1, 2, and 28).

### Statistical Analysis

All analysis was done using R Statistical Software (R Core Team, 2022). A stimulus was categorized as “positive” or “negative” if its mean valence rating from the survey was significantly greater than or less than zero, respectively, at the p < 0.00125 significance level (α = 0.05 after Bonferroni correction for 40 comparisons). A stimulus was considered “neutral” if its mean valence rating was not significantly different from zero. This categorization scheme yielded 11 faces in the “negative” valence category, 15 in the “neutral” category, and 14 in the “positive” category (see Appendix 1).

### Study 2. Emotion Recognition in Adolescents with ASD Compared to TD Adolescents

Study participants were administered both RMET-A and RMET-C. Emotional valence data from the above survey were used to examine whether performance on these ToM tasks was influenced by the emotional valence of the test items. Participants were not aware of valence ratings, and the order of presentation was the standard order in the originally published RMET test descriptions. The RMET-C was administered prior to administration of the RMET-A for all participants.

### Participants

Participants were recruited through clinics at the University of Massachusetts Medical Center, postings on the []Child and Adolescent Neurodevelopment Initiative (CANDI) website, the Eunice Kennedy Shriver Center website, Social Media posting/outreach, department newsletters, and via word of mouth and email to providers working with adolescents. All recruitment materials and/or text were approved by the UMass Chan Institutional Review Board prior to use.

Parents of participants provided written informed consent. Participants provided written assent.

Inclusion criteria: Adolescents 13–17 years of age with a Full-Scale Intelligence Quotient (FSIQ-2) greater than 70 as estimated by the Vocabulary and Matrix Reasoning subtests of the Wechsler Abbreviated Scale of Intelligence (WASI) (Wechsler,^1999^).

Exclusion criteria included FSIQ-2 less than 70; major medical or neurological illness; unstable psychiatric illness interfering with ability to complete study; clinically significant suicidality.

Diagnosis in the ASD group was by criteria from The Diagnostic and Statistical Manual of Mental Disorders (5th ed.; DSM–5; American Psychiatric Association, 2013). This was accomplished through a clinical interview with a child and adolescent psychiatrist and administration of the Autism Diagnostic Observation Scale (ADOS-2) (Lord et al.,^1999^) and the Autism Diagnostic Interview-Revised (ADI-R) (Rutter et al., 2003) by a research-reliable clinician. Psychiatric comorbidities were assessed by clinical interview with a child and adolescent psychiatrist.

### Measures

#### SRS-2

The Social Responsiveness Scale, 2nd edition (SRS-2), a questionnaire that measures the severity of social impairment in ASD, was administered to the primary caregivers of all subjects. The SRS-2 is a quantitative measurement of various dimensions of interpersonal behavior, communication, and repetitive/stereotypic behaviors, including subscales for Social Awareness, Social Cognition, Social Communication, Social Motivation, and Restricted Interests/Repetitive Behaviors (Constantino, 2012). Higher scores reflect greater deficits in each area.

#### RMET

The adult and the child versions of the RMET were used to assess ToM abilities among the adolescents. The RMET-A consists of 37 (one practice and 36 test) black and white photographs of the eye region of various faces, and the RMET-C consists of 29 (one practice and 28 test) such photographs. All but three photographs used in the RMET-C are also part of the RMET-A stimuli. In both versions, four possible emotion labels (three foils and one target response) surround each face; however, the vocabulary used in the RMET-A is more sophisticated than that used in the RMET-C. Participants completed paper-and-pencil versions of both the RMET-A and the RMET-C with no time limit. From four labels (three foils and one correct response), participants identified the option that they believed most accurately describes the emotion that the face is expressing.

### Statistical Analysis

The R Statistical programming language (https://www.r-project.org) was used for all analyses (R Core Team, 2022), along with the contributed packages *emmeans* (Lenth et al., 2022), *ggplot2* (Wickham, 2016), and *nlme* (Pinheiro & Bates, 2000; Pinheiro et al., 2022). Within the Child and Adult versions of the RMET, a linear mixed effects model was specified with Error Rate as the dependent variable and Group (ASD, HC) and Valence (NEGATIVE, NEUTRAL, POSITIVE) as independent variables (as main effects and their interaction), and a random effect of subjects. Post-hoc comparisons compared the overall error rate by group, the error rate by valence category, and the group effect within each valence category. Exploratory analyses extended the model by the inclusion of RMET version as a main effect. False discovery rate was controlled using the method of Benjamini and Hochberg (Benjamini & Hochberg, 1995).

## Results

### Study 1. Identifying Emotional Valence of RMET Photographs

One hundred thirteen medical school students (45 male, 68 female) with an age range of 22 to 42 years (Mean = 26.0 years) completed the survey to classify emotional valences of the eye region photographs. Across all faces from both the RMET-A and RMET-C, the assigned valences had a narrow spread across a range of 3.43, with potential range up to 7.0. Of the 36 test items in the RMET-A, 11 (30.5%) were assigned a negative valence, 14 (39%) were assigned a positive valence, and 11 (30.5%) were assigned a neutral valence. Of the 28 test items in the RMET-C, 10 (36%) were assigned a negative valence, 9 (32%) were assigned a positive valence, and 9 (32%) were assigned a neutral valence.

### Study 2. Emotion Recognition in ASD vs. TD

A total of 33 subjects with ASD and 30 TD adolescents participated in the study (see Table 1 for demographics).

**Table 1:** Characteristics of the participants

	TD	ASD	p
	n = 30	n = 33	
Sex (Male (%))	25 (83.3)	31 (93.9)	0.349
Age (mean (SD))	15.05 (1.63)	14.81 (1.43)	0.533
Race (n, %)			0.279
White	22 (78.6)	29 (90.6)	0.279
Black/African-American	0 (0.0)	1 (3.1)	
Multiracial	2 (7.1)	1 (3.1)	
Other/Unknwn	4 (14.3)	1 (3.1)	
Ethnicity (n, %)			
Hispanic/Latinx	4 (16.7)	2 (6.2)	0.418
FSIQ (mean (SD))	116.96 (14.02)	109.61 (15.85)	0.062
SRS-2 (raw scores, mean(SD))			
Total Score	19.47 (15.29)	86.58 (28.57)	< 0.001
Social Awareness	4.50 (2.78)	11.39 (3.22)	< 0.001
Social Cognition	3.00 (3.72)	16.06 (6.31)	< 0.001
Social Communication	5.70 (5.38)	30.27 (11.22)	< 0.001
Social Motivation	4.60 (3.70)	13.42 (6.06)	< 0.001
Restricted Repetitive Behaviors	1.67 (1.99)	15.42 (6.12)	< 0.001
SRS-2 (T scores, mean (SD))			
Total Score	44.57 (6.09)	71.18 (11.27)	< 0.001
Social Awareness	45.83 (7.59)	68.09 (10.08)	< 0.001
Social Cognition	43.87 (6.15)	68.48 (11.61)	< 0.001
Social Communication	44.33 (5.44)	71.70 (12.03)	< 0.001
Social Motivation	47.17 (7.55)	65.48 (12.50)	< 0.001
Restricted Repetitive Behaviors	44.23 (3.59)	68.67 (10.90)	< 0.001

ASD = Autism Spectrum Disorder; FSIQ = Full-Scale Intelligence Quotient-2 from Weschler Abbreviated Scale of Intelligence, 2nd edition; SRS = Social Responsiveness Scale; SD = Standard Deviation; TD = Typically Developing

**Fig. 1:**
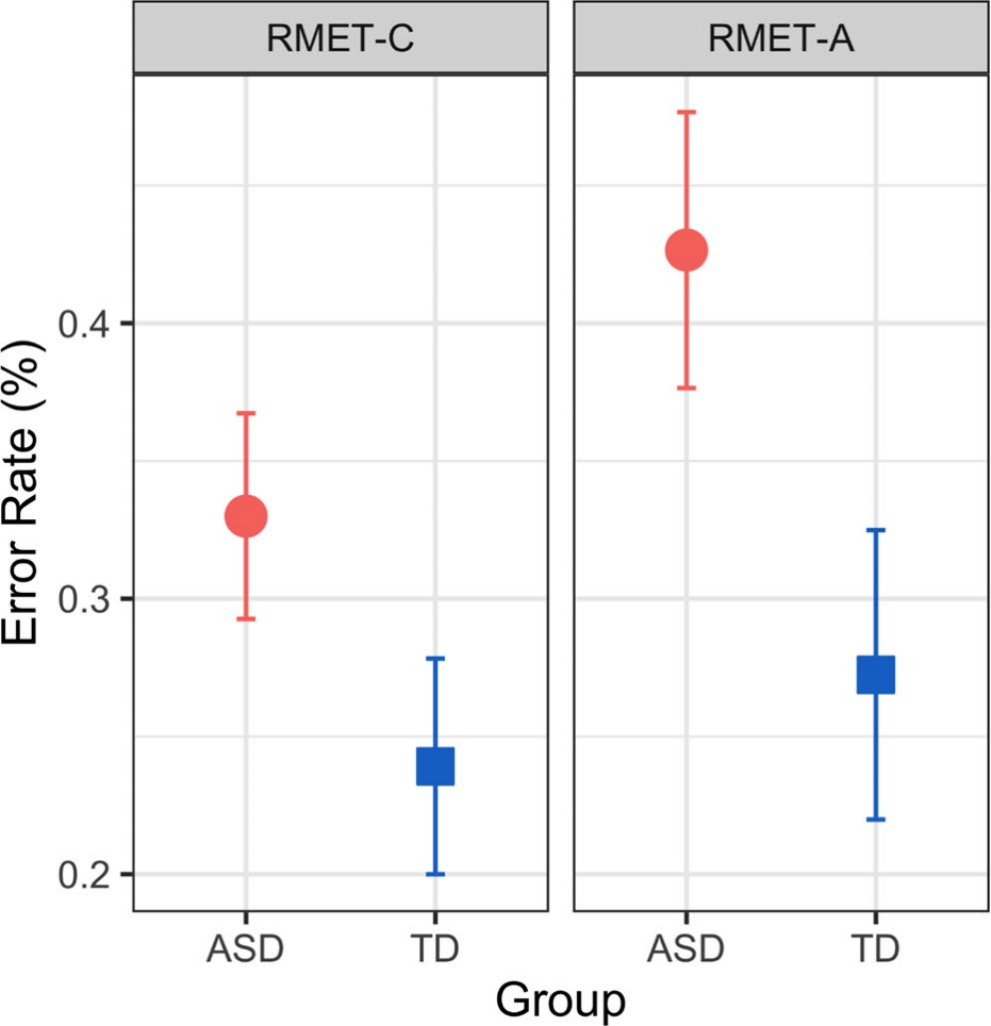
RMET error rate by group (ASD and TD) and test version (RMET-A and RMET-C)

The ASD group had a significantly higher average error rate on both the RMET-A and RMET-C compared to the TD group (Fig. 1). On the RMET-A, the ASD group had an error rate of 42.7% (95% Confidence interval: 37.6–47.7%) compared to 27.2% (22.0–32.5%) for the TD group (t(65.1) = -4.2, p < 0.0001). On the RMET-C, the ASD group had an error rate of 33.0% (29.3–36.7%) compared to 23.9% (20.0–27.8%) for the TD group (t(65.1 = -3.4, *p* = 0.0013).

**Fig. 2:**
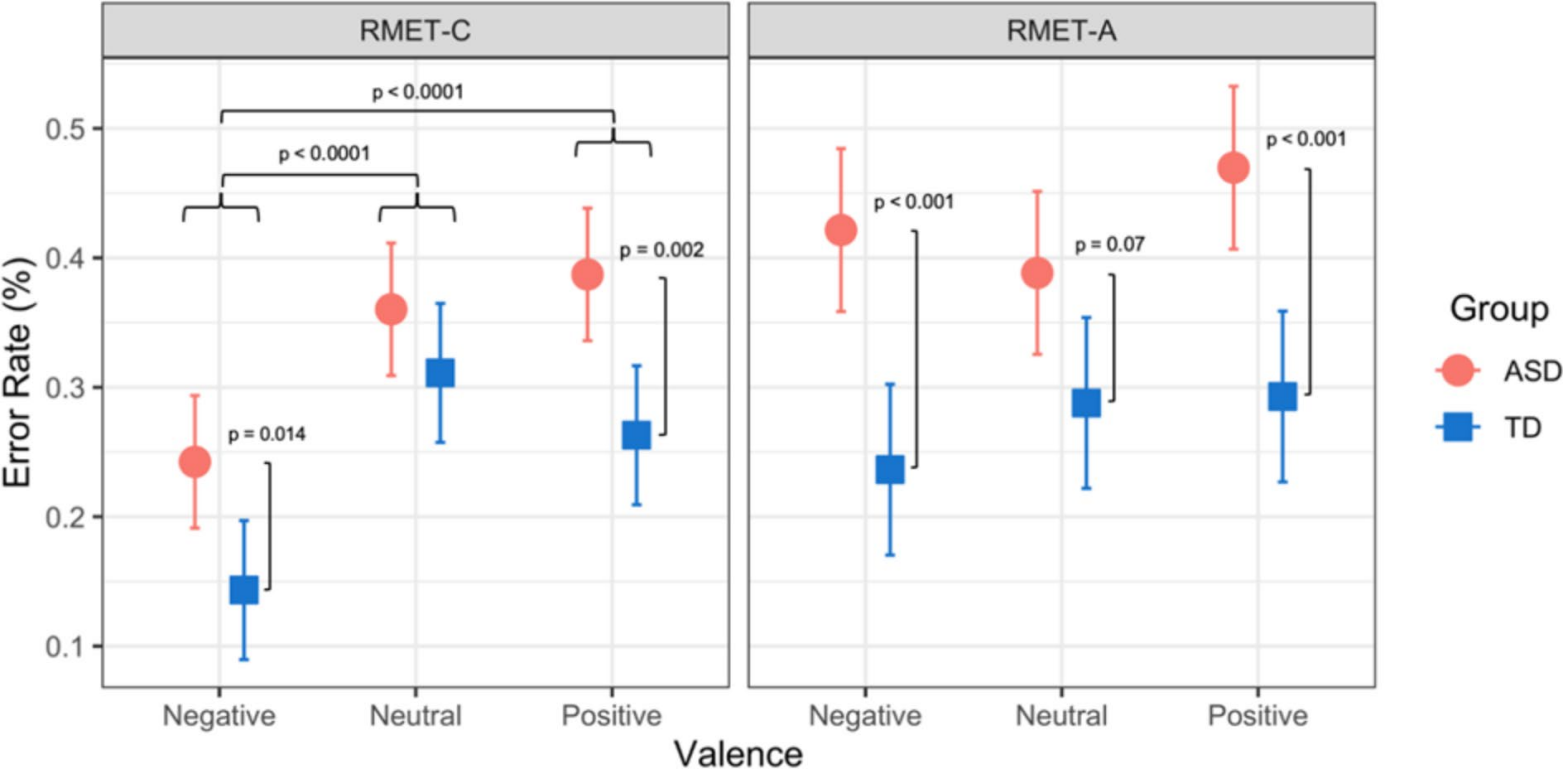
RMET error rate by group (ASD and TD), test version (RMET-A and RMET-C) and emotional valence (Negative, Neutral, Positive)

In order to determine the effects of valence and group on RMET performance, analysis was done to quantify the effect of interaction between these variables on error rates of the RMET-A and RMET-C (Fig. 2). On the RMET-A, error rates did not significantly differ by valence within either the ASD (range = 38.8–47.0%, all p > 0.4) nor the TD (range = 23.6–29.3%, all p > 0.3) groups. Between the ASD and TD groups, the difference in error rate for a given valence was more pronounced for positive valence (mean difference = -17.7%, t(142) = -3.8, p = 0.0009) and negative valence (mean difference = -18.5%, t(142) = -4.0, p = 0.0009) than for neutral valence (mean difference = -10.1%, t(142) = -2.1, p = 0.07).

On the RMET-C, the TD group’s error rate for negative valence (14.3%, CI 9.0–19.7%) was significantly lower than that of neutral (31.1%, CI 25.7–36.5%; mean difference = -16.8%, t(130) = -5.1, p < 0.0001) and positive valence (26.3%, CI 20.9–31.7%; mean difference = -12.0%, t(130) = -3.7, p = 0.001). There was not a significant difference in error rate between neutral and positive valence (mean difference = 4.8%, t(130) = 1.5, p = 0.3). Results were analogous for the ASD group: error rate for negative valence (24.2%, CI 19.1–29.4%) was significantly lower than that of neutral (36.0%, CI 30.9–41.1%; mean difference = -11.8%, t(130) = -3.8, p = 0.0007) and positive valence (38.7%, CI 33.6–43.8%; mean difference = -14.5%, t(130) = -4.6, p < 0.0001), and there was not a significant difference in error rate between neutral and positive valence (mean difference = -2.9%, t(130) = -0.9, p = 0.7). Between the ASD and TD groups, the difference in error rate for a given valence was more pronounced for positive valence (mean difference = -12.4%, t(169) = -3.3, p = 0.002) than for negative valence (mean difference = -9.9%, t(169) = -2.6, p = 0.01) or neutral valence (mean difference = -4.9%, t(169) = -1.3, p = 0.2).

Each group’s performance was also compared across RMET versions for each valence level. The ASD group had a significantly higher error rate for negative valence items (mean difference = 17.9%, t(305) = 5.2, p < 0.0001) and positive valence items (mean difference = 8.2%, t(305) = 2.4, p = 0.04) on the RMET-A compared to the RMET-C, but not neutral valence items (mean difference = 2.8%, t(305) = 0.8, p = 0.5). The TD group made fewer errors on the negative valence items in the RMET-C (mean difference = 9.3%, t(305) = 2.6, p = 0.3), but did not perform significantly differently on neutral or positive valence items between the RMET versions (mean difference range = -2.3 − 3.0%, all p > 0.5).

## Discussion

### Summary of Findings

We first determined the emotional valence of each eye region stimulus from the RMET-C and RMET-A. On the RMET-C, 36% of items were classified as negative valence with the rest evenly split between positive and neutral valence. On the RMET-A, 39% were classified as positive valence with the rest evenly split between negative and neutral valence.

We next compared performance of ASD and TD groups on both the RMET-A and RMET-C and quantified how emotional valence impacted this performance. Results showed that adolescents with ASD had higher error rates compared to TD on both the RMET-C and RMET-A. While adolescents in the ASD group consistently demonstrated higher error rates than TD with all stimuli regardless of valence and in both versions of the RMET, differences between groups were greater with positive and negative valence items than with neutral items. Both groups had the lowest error rate when performing the RMET-C test with negative valence stimuli. Both groups performed better overall on the RMET-C compared to the RMET-A; however, this difference was more pronounced in the performance of individuals with ASD.

### Comparison to Literature – Valence Classification of RMET Eye Regions

Multiple approaches to emotional valence classification of the RMET stimuli have been used. Hudson and colleagues (2020) outline these methodological variations and show how such inconsistencies, together with varying sample sizes, make it challenging to draw conclusions about how valence may affect RMET performance. Of these previous studies, we compared our emotional valence classification system to the four most commonly used (Koizumi & Takagishi 2014, Baribeau et al. 2015, Harkness et al. 2005, Scott et al. 2011). The first two of these studies applied emotional valence classification to the RMET-C while the second two applied emotional valence classification to the RMET-A. We compared how consistent our valence classification was to those published in the existing literature and discuss the impact of methods used to establish classifications.

**Fig. 3:**
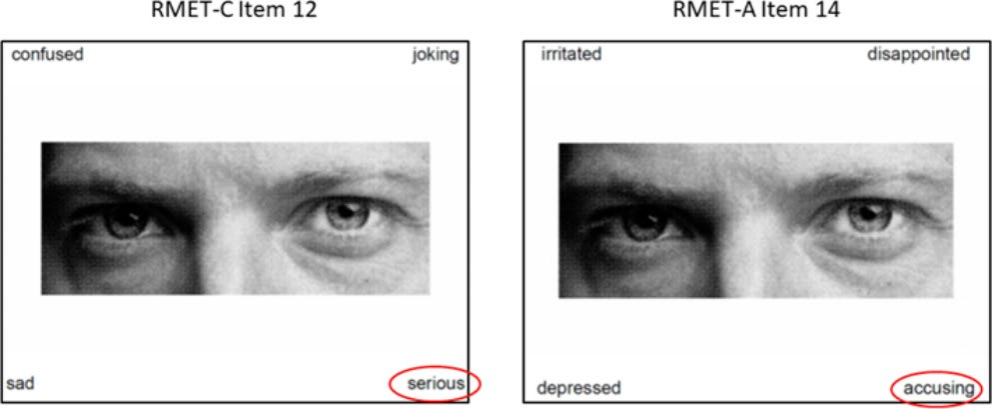
Comparison of corresponding items from RMET-A and RMET-C with conflicting valence classifications in previous studies in which correct label was presented alongside the photograph

Our RMET-C valence classifications have a 57% agreement with those of Koizumi & Takagishi (2014) and a 54% agreement with Baribeau and colleagues (2015) (Appendix 1). Koizumi & Takagishi (2014) state that items were classified by their emotional valence but do not indicate the method used for their classification. They cite Fertuck and colleagues (2009), who in turn used the classifications of Harkness and colleagues (2005) on the 36-item RMET-A, which may not be directly applicable to the 28-item RMET-C. Also, there is only 56% agreement between the classification of overlapping items in Koizumi & Takagishi (2014) and Harkness et al. (2005). One possible explanation for these discrepancies is that similar to Hudson et al. (2020), both studies included the “correct” emotion label with the photograph during the classification task and, therefore, may have imparted bias to the subjects. Since the correct labels differ between the two RMET versions, the potential for bias can be seen in Fig. 3, which displays an item that was classified by Koizumi & Takagishi as “neutral” (correct label is “serious” in the RMET-C), but that same item was classified by Harkness and colleagues as being “negative” (correct label is “accusing” in the RMET-A). By removing the labels and having subjects classify the valence using only the image of the eye region, our study addresses the potential bias associated with the label.

Baribeau and colleagues (2015) derived their own RMET-C valence classifications from Koizumi & Takagishi (2014) and two differing RMET-A classifications (Harkness et al.,2005; Scott et al., 2011)). They assigned the valence by consensus of the other three study assignments, choosing a neutral valence for the two items that were assigned to three differing categories by the three prior studies (see Appendix 1 – RMET-C Items 17 and 26, RMET-A Items 19 and 34). Thus, their classification scheme suffered from a combination of the weaknesses of the three prior studies.

**Fig. 4:**
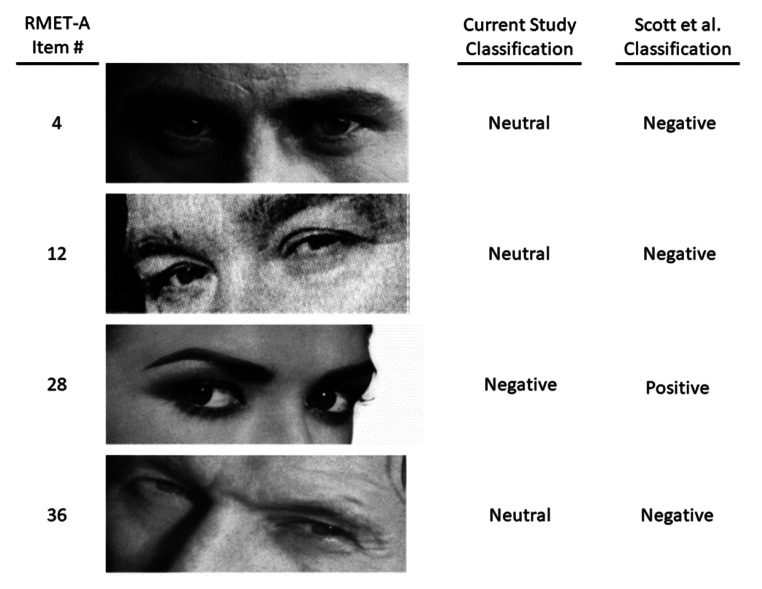
Comparison of RMET items with differing valence classifications between present study and Scott et al. (2011)

Our RMET-A valence classifications have a 44% agreement with the study by Harkness and colleagues (2005) and a 64% agreement with the study by Scott and colleagues (2011) (Appendix 1). While the valences used in the study Harkness and colleagues were determined by raters who were shown each face together with its correct label, valences in the study by Scott and colleagues were determined independently of the labels, likely explaining the greater agreement with the present study. The participants who classified the stimuli also differed between the studies: Harkness and colleagues surveyed 12 undergraduate women, while Scott and colleagues surveyed 40 undergraduate students (males and females), and in the present study we surveyed 113 medical school students (45 male, 68 female). Due to the differences in sample sizes, it is possible that the study with the lower number of individuals surveyed led to some stimuli being classified as neutral due to not meeting the significance threshold (Type 2 error). Most of the differences (9/13 items; 69%) in classifications between our study and the study by Scott and colleagues (2011) could be accounted for by decreased power to detect significant positive or negative valence in the earlier study. It is possible that the age, clinical experience and training of the medical students used for valence classification in our study contributed to the different classifications of the remaining 4 items between these two otherwise similar studies. Figure 4 shows the items with disagreement, not explained by insufficient power in the previous study, along with the assigned valence in our study and in the study by Scott and colleague. Further clinical assessments were not performed on these convenience samples, so it is unclear if differences in social cognition between these groups contributed to the differing classification results.

### Comparison to Literature – Emotional Recognition in TD and ASD by Valence

Individuals with ASD had a higher error rate on both versions of the RMET compared to TD, which is consistent with previous ASD RMET studies (Baribeau et al., 2015; Demurie et al., 2011; Kaland et al., 2008) and a substantial body of research suggesting impaired ToM in ASD (Capps et al., 2000; Happe et al., 1996; Harms et al., 2010; Lartseva et al., 2015). Individuals with ASD performed significantly worse on positive items compared to neutral and negative items in a prior study by Baribeau and colleagues (2015). In contrast, we found individuals with ASD performed significantly worse on both positive and neutral items compared to negative on the RMET-C, and they performed slightly worse on the RMET-A overall. This was likely a result of our differing valence classifications, given that almost half of the eye regions were classified differently in our present study. Given the methodological limitations of the previously used classification schemas outlined above, the present study may be a more accurate reflection of emotion recognition accuracy by valence.

Our findings differ from those of non-RMET valence studies that suggest subjects with ASD have greatest difficulty identifying emotions of negative valence (Ashwin et al., 2006; Howard et al., 2000; Pelphrey et al., 2002; Wallace et al., 2011). Much of this difference likely stems from these studies’ use of different experimental paradigms, consisting of far fewer total emotions than either RMET version with a greater proportion being negative (fear, sadness, disgust, and anger) (Howard et al., 2000; Pelphrey et al., 2002; Wallace et al., 2011).

Similar to the present study, Kaland and colleagues (2008) studied performance on both the RMET-C and RMET-A in TD adolescents and in individuals with ASD. Both groups showed a higher accuracy on the child version than the adult version; however, the authors did not report on the significance of the differences between versions in comparing each group’s performance.

### Significance of Findings & Future Directions

In our classification of RMET stimuli, we found a relatively narrow valence range across all images. In a study of facial emotion recognition, Wallace and colleagues (2011) demonstrated that adolescents with ASD require more intense facial expressions for accurate emotion identification compared to TD controls. Since our stimuli did not have a high intensity of valence, this supports the ability of the RMET to detect more subtle difficulties with emotion recognition in ASD. However, it is necessary to establish validated valence classifications for stimuli across both RMET versions, as the classifications to date show limited concordance. We believe that the strengths of our valence classification method compared to previous reports, including increased sample size and identification of valence without biasing results by including “correct” labels, make our classification results more reliable. However, future validation of the results by replication is warranted.

Interestingly, while individuals with ASD consistently performed worse compared to TD, RMET-A performance did not significantly differ by valence within either group. By contrast, in the RMET-C both groups performed better at identifying negative valence emotions. Negative emotional stimuli tend to capture more attentional resources compared to positive emotional stimuli, which may explain this trend (Balconi et al., 2012; Charles et al., 2003; Johansson et al., 2004; Öhman et al., 2001; Rumpfa et al., 2012). Our study demonstrates that impairment in the ability of individuals with ASD to identify emotions is primarily seen in identification of positive or negative valence emotions, with no difference between groups on identification of neutral items. The increased language complexity of the RMET-A, meanwhile, further compounded the increase in error rate when individuals with ASD were asked to identify positive or negative emotions. This suggests that a task involving processing of complex language interacts with the emotional demands required to identify extremes of emotional valence, exacerbating the challenge that individuals with ASD experience when interpreting nonverbal emotional cues. This finding has clinical implications, as individuals with ASD may be more significantly impacted by their social communication deficits when combined with demands that stretch their verbal language processing capacity.

### Limitations

The present study should be interpreted in light of the following limitations. Due to the low number of subjects, we may have lacked the power needed to identify significant differences in RMET performance, especially in the TD group where differing performance among valences and between RMET versions was more subtle. Given the results in the TD group on RMET-A, we estimate that a prospective study would need to include nearly 3 times as many TD participants (n = 90) in order to have 80% power to detect a difference in error rate at least as large as observed here between the valences. Another limitation was the IQ difference between groups, which may contribute to the higher discrepancy between groups in the RMET-A. However, both groups had above average IQ, and the difference between groups was not statistically significant at the α = 0.05 level. Further study with more subjects that are matched more closely for IQ will help to clarify these results.

## Conclusion

The present study investigated how the emotional valence of testing stimuli might impact performance on the RMET ToM task for a population of adolescents with ASD and TD. The valence of each face stimulus from the RMET-A and RMET-C was first classified as “negative,” “neutral,” or “positive.” While these classifications had a variable degree of agreement with those in the literature, our methods for determining these classifications had less potential for bias. Adolescents with ASD demonstrated impaired ToM on the RMET-A and -C compared to TD. When less complex emotional language was used, individuals in both groups performed best. This trend, however, was more pronounced among adolescents with ASD. Therefore, the RMET-C may be best in this age range to identify pure emotion recognition deficits, but the RMET-A may be superior in highlighting discrepancies seen when other competing cognitive demands are present. The present study provides specific methods for classifying the emotional valence of stimuli for studies of the RMET, and the mean valences and classifications (Appendix 1) provided in the present study will allow for more nuanced future study of emotion recognition by valence in adolescents with ASD as well as TD and other clinical populations when using this test.

## Appendix 1

**Table A1:** Comparison of Valences from Different RMET Classification Systems

RMET-C Item Number, if applicable (RMET-A item, if applicable)	Correct Valence Label, RMET-C (Correct Valence Label, RMET-A)	Valence (Present Study) Mean (95% CI)	Valence (Baribeau et al. 2015)	Valence (Koizumi & Takagishi 2014)	Valence (Scott et al. 2011)	Valence (Harkness et al. 2005)
1	Kind	Neutral 0.12 (-0.04, 0.29)	Positive	Positive		
2	Sad	Negative -0.5 (-0.67, -0.34)	Negative	Negative		
3 (1)	Friendly (Playful)	Positive 0.66 (0.38, 0.95)	Positive	Positive	Positive	Positive
4 (2)	Upset (Upset)	Negative -1.64 (-1.86, -1.42)	Negative	Negative	Negative	Negative
(3)	(Desire)	Positive 0.56 (0.37, 0.74)			Neutral	Neutral
5 (4)	Making somebody do something (Insisting)	Neutral -0.12 (-0.28, 0.03)	Neutral	Neutral	Negative	Neutral
6 (5)	Worried (Worried)	Negative -1.06 (-1.25, -0.88)	Negative	Negative	Negative	Negative
(6)	(Fantasizing)	Positive 0.59 (0.4, 0.79)			Neutral	Positive
7 (7)	Interested (Uneasy)	Positive 0.43 (0.25, 0.62)	Neutral	Positive	Neutral	Neutral
8 (8)	Remembering (Despondent)	Negative -1.06 (-1.24, -0.89)	Neutral	Neutral	Negative	Neutral
9 (9)	Thinking about something (Preoccupied)	Positive 0.32 (0.17, 0.47)	Neutral	Neutral	Neutral	Neutral
(10)	(Cautious)	Positive 1.18 (0.97, 1.38)			Neutral	Neutral
(11)	(Regretful)	Neutral 0.19 (0.03, 0.35)			Neutral	Negative
10 (12)	Not believing (Sceptical)	Neutral 0.37 (0.14, 0.6)	Negative	Negative	Negative	Neutral
11 (13)	Hoping (Anticipating)	Positive 0.71 (0.49, 0.93)	Positive	Positive	Positive	Neutral
12 (14)	Serious (Accusing)	Negative -0.47 (-0.67, -0.26)	Negative	Neutral	Negative	Negative
13 (15)	Thinking about something (Contemplative)	Positive 0.67 (0.49, 0.85)	Neutral	Neutral	Positive	Neutral
14 (16)	Thinking about something (Thoughtful)	Neutral 0.24 (0.09, 0.38)	Neutral	Neutral	Neutral	Positive
15 (17)	Not believing (Doubtful)	Neutral -0.1 (-0.28, 0.08)	Negative	Negative	Neutral	Negative
16 (18)	Made up her mind (Decisive)	Neutral -0.1 (-0.34, 0.14)	Neutral	Neutral	Neutral	Neutral
17 (19)	A bit worried (Tentative)	Positive 0.67 (0.48, 0.87)	Neutral	Negative	Positive	Neutral
(20)	(Friendly)	Positive 1.68 (1.52, 1.84)			Positive	Positive
(21)	(Fantasizing)	Positive 1.46 (1.26, 1.66)			Positive	Positive
(22)	(Preoccupied)	Negative -0.91 (-1.07, -0.75)			Neutral	Negative
(23)	(Defiant)	Negative -0.61 (-0.78, -0.44)			Neutral	Negative
18 (24)	Thinking about something sad (Pensive)	Negative -0.35 (-0.54, -0.17)	Neutral	Negative	Neutral	Neutral
19 (25)	Interested (Interested)	Positive 0.85 (0.62, 1.08)	Positive	Positive	Positive	Positive
20 (26)	Not pleased (Hostile)	Negative -1.75 (-1.92, -1.58)	Negative	Negative	Negative	Negative
(27)	(Cautious)	Neutral -0.15 (-0.31, 0.01)			Neutral	Negative
21 (28)	Interested (Interested)	Negative -0.36 (-0.57, -0.16)	Positive	Positive	Positive	Neutral
22 (29)	Thinking about something (Reflective)	Neutral -0.04 (-0.21, 0.14)	Neutral	Neutral	Neutral	Neutral
(30)	(Flirtatious)	Positive 1.04 (0.82, 1.27)			Neutral	Positive
23 (31)	Sure about something (Confident)	Neutral -0.19 (-0.4, 0.01)	Neutral	Neutral	Neutral	Positive
24 (32)	Serious (Serious)	Negative -1.63 (-1.82, -1.43)	Neutral	Neutral	Negative	Neutral
25 (33)	Worried (Concerned)	Negative -1.04 (-1.2, -0.89)	Negative	Negative	Negative	Neutral
26 (34)	Nervous (Distrustful)	Positive 0.82 (0.62, 1.03)	Neutral	Neutral	Positive	Negative
(35)	(Nervous)	Neutral -0.29 (-0.48, -0.11)			Neutral	Negative
27 (36)	Not believing (Suspicious)	Neutral -0.41 (-0.74, -0.08)	Negative	Negative	Negative	Negative
28	Happy	Positive 1.65 (1.47, 1.84)	Positive	Positive		
